# Prevention is better than treatment 

**DOI:** 10.2471/BLT.15.020915

**Published:** 2015-09-01

## Abstract

Developing countries face a growing toll of tooth decay and gum disease that can be prevented. Apiradee Treerutkuarkul and Karl Gruber report.

In the early 2000s, Thailand was in the throes of rolling out universal health care for its citizens and Prathip Phantumvanit knew – from years of dental practice and research – that tooth decay was remarkably widespread among adults and children for a developing country.

Phantumvanit teamed up with the health ministry to create a new cadre of dental nurses to staff rural health centres and community hospitals. 

These nurses became key to running an outreach programme for the prevention of tooth decay and gum disease.

“There were few dentists in our country and most people could not afford dental services,” recalls Phantumvanit, who founded the faculty of dentistry at Thammasat University in Bangkok, where he is a professor. “These dental nurses worked well with the appropriate equipment and technology to fulfil this need”.

“There were few dentists… and most people could not afford dental services.”

Prathip Phantumvanit

A core element of the Thai oral health programmes has been promoting tooth-brushing with fluoridated toothpaste in primary schools – health education made possible by collaborating with teachers and parents. 

The result: the proportion of children with tooth decay in their permanent teeth has decreased and children, parents and schoolteachers are more aware of the importance of oral health, according to the 2013 national survey on oral health. 

Diseases of the mouth, such as tooth decay or cavities (dental caries) and gum disease (periodontal disease) are among the most common noncommunicable diseases in the world and, traditionally, some of the most neglected. 

“The incidence of tooth decay in low- and middle-income countries is rapidly increasing among adults and children and there will be a huge burden of this health problem in the future without sustainable prevention programmes,” says Professor Poul Erik Petersen, director of the World Health Organization (WHO) Collaborating Centre of Community Oral Health and Research at the University of Copenhagen in Denmark.

Tooth decay affects an estimated 60–90% of schoolchildren and nearly 100% of adults worldwide, according to the WHO Global Oral Health Database. 

Severe dental disease can result in tooth loss and the prevalence of complete tooth loss is increasing rapidly in low- and middle-income countries, while currently about 30% of the world’s population aged 65–74 lose all their natural teeth. 

According to a recent Global Burden of Disease study, untreated tooth decay is the most prevalent of 291 major diseases and injuries. Periodontal disease is the sixth most prevalent.

“If left untreated, dental diseases can cause severe pain, infection and negatively impact the quality of life, children’s growth, school attendance and performance, and can lead to poor productivity at work and absenteeism in adults,” says Wagner Marcenes, director of research at Barts Health NHS Trust in London, the United Kingdom of Great Britain and Northern Ireland, who led the Oral Health Research Group within the Global Burden of Disease study.

“The fact that the burden of preventable tooth decay and gum disease is increasing … should serve as a wake-up call for policy-makers to recognize the importance of dental health,” says Marcenes.

 In response to the growing toll of oral disease, high-income countries rely on treatment, the cost of which often falls on individuals, while prevention measures such as water fluoridation over the last 30 years have improved the situation in some of these countries. 

For low- and middle-income countries, meeting the current need for treatment would exceed most health-care budgets, leaving prevention as the only viable option. 

“WHO is working with countries to develop policies to prevent oral health problems: so that fewer people develop tooth decay, gum disease and other oral diseases in the first place,” says Dr Hiroshi Ogawa, a dental officer at WHO. 

 The key factors driving the epidemic of tooth decay are the increasing consumption of sugary foods and drinks and the inadequate use of fluoridated toothpaste, water, salt and milk, to prevent tooth decay. 

“It’s primarily what we call *free sugars* – the sugars added to foods by manufacturers, cooks and consumers – that are causing tooth decay,” says Professor Paula Moynihan, who runs a WHO Collaborating Centre for Nutrition and Oral Health at Newcastle University in the United Kingdom. 

“In many industrialized countries the diet includes an increasing array of food products with a high – and often hidden – sugar content, such as breakfast cereals, pretzels and ketchup,” says Moynihan, the principal investigator in one of the systematic reviews that provided the scientific basis for WHO’s revised guidelines on free sugars’ intake that were released in March.

In 2003, a WHO guideline recommended that individuals should restrict their intake of free sugars to less than 10% of daily caloric intake. The recommendation was hotly contested at the time by the food industry. 

A decade later the scientific evidence that had since emerged not only reinforced the 2003 recommendation, but supported reducing free sugars to less than 5% of daily caloric intake to reap additional health benefits, including reducing tooth decay.

But for seasoned public health promoters, like Dr Noeline Razanamihaja in Madagascar, the key finding of Moynihan and colleagues’ systematic review – that the more sugar people consume, the more tooth decay they have – was not a surprise. 

“In Madagascar we often consume food and drink containing free sugars and traditionally we did not brush our teeth regularly,” says Razanamihaja, a professor at Mahajanga University. 

“As a result, tooth decay has been extremely high among our children, many of them have tooth ache and this affects school hours and teaching,” says Razanamihaja, who helped to set up a programme for oral disease prevention in Madagascar in 1996. 

“We managed to make this a sustainable programme by building a strong collaboration with all parties involved: the health ministry, public health officials, dentists, teachers, communities, schools and families,” she says, adding: “Families support the programme in schools and make small financial contributions towards it. Toothbrushes and toothpaste are locally produced and reasonably priced and surveys have shown that since 1996 we have better oral health and better oral health awareness in our population”.

To address the high toll of tooth decay and gum disease, many high-income countries still rely largely on treatment – which accounts for between 5% and 10% of their health expenditure – rather than prevention. 

Treatment, however, is not an option for most people in low- and middle-income countries, due to the expense and lack of dentists. At the same time, the consumption of sugary foodstuffs and drinks is increasing in developing countries.

“In low- and middle-income countries the prevalence of tooth decay is escalating and, in most cases it is left untreated or tooth extraction takes place in case of pain or other symptoms,” says Petersen, who led the WHO global oral health programme for 12 years. 

“These countries do not have adequate economic resources to meet the need for dental treatment and that is why we need oral health systems oriented towards disease prevention and health promotion,” he says.

“We need oral health systems oriented towards disease prevention and health promotion”.

Poul Erik Petersen

In 2003, WHO highlighted four essential oral health messages for young children and families to be reinforced through health education projects, such as the ones in Madagascar and Thailand: brush your teeth twice a day, use effective fluoridated toothpaste, consume fewer sugary food and drinks each day and eat more fruit and vegetables. 

The prevention of oral and other noncommunicable health problems will also require stronger food policy in many countries, including “…food and nutrition labelling, consumer education, regulation of marketing of food and non-alcoholic beverages that are high in free sugars, and fiscal policies targeting foods that are high in free sugars”, according to the revised WHO guideline entitled *Sugars intake for adults and children.*

And, while there is abundant scientific evidence that regular tooth-brushing with fluoridated toothpaste helps to prevent tooth decay, most developing countries do not provide affordable toothpastes or toothbrushes, not to mention fluoridated water, salt or milk. 

In a 2008 study of the global affordability of fluoridated toothpaste, Dr Habib Benzian, who teaches at the College of Dentistry, New York University in the United States of America, and his colleagues found striking differences in the annual household expenditure needed to purchase a year’s supply of fluoridated toothpaste, ranging from 0.02% in the United Kingdom to 4% in Zambia. 

Moreover, in low- and middle-income countries fluoridated toothpaste is often of poor quality. Some middle-income countries manufacture their own toothpaste but the level of fluoride is sometimes too low to be effective and national regulations or even international quality standards are not observed or enforced.

“Even though toothpaste is widely available for purchase, the cost, particularly for the poor and disadvantaged, is a major barrier for regular use,” says Benzian.

Nongovernmental organizations, such as the Global Children’s Oral Health and Nutrition Program, are working in several developing countries in Latin America and Asia and India Smiles in India holds dental camps to provide toothbrushes, toothpaste and oral hygiene education. 

 For WHO’s Ogawa such projects are commendable “but bring relief to relatively few people, and cannot reverse the coming plague of tooth decay and gum disease sweeping these countries”. 

**Figure:**
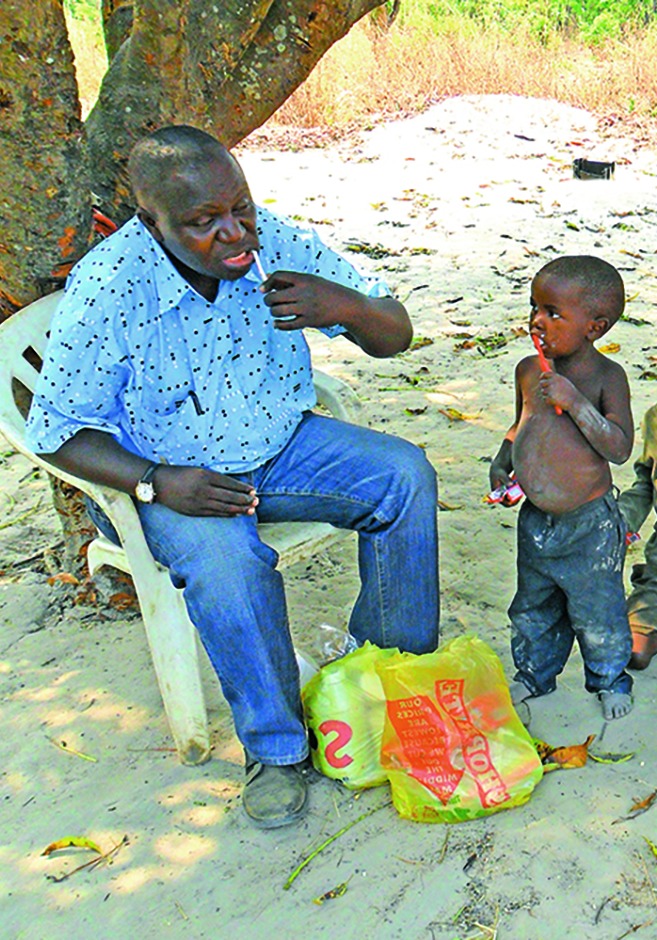
Elastus Chonde, a dental project manager, teaches children how to brush their teeth. Chonde was working on Global Child Dental Fund’s 2012 Smiles and Hope Zambia project. The project targeted 1000 disadvantaged families in Zambia.

© Global Child Dental Fund

**Figure:**
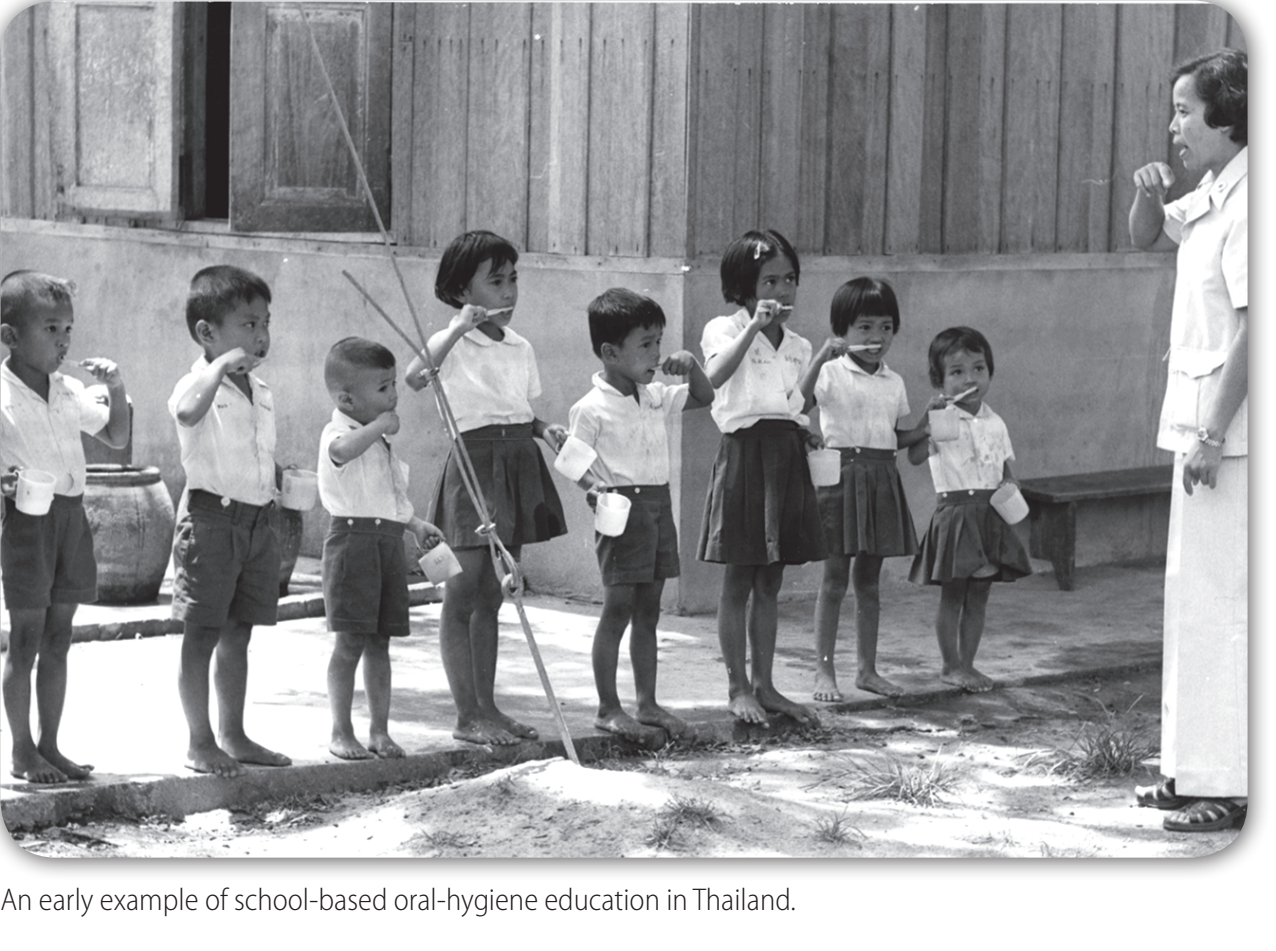
An early example of school-based oral-hygiene education in Thailand.

WHO

